# Investigating the Effect of Trace Levels of Manganese Ions During Solvothermal Synthesis of Massey University Framework-16 on CO_2_ Uptake Capacity

**DOI:** 10.1021/acs.chemmater.4c00137

**Published:** 2024-05-17

**Authors:** Akriti Sarswat, John Bacsa, Ankana Roy, Joao Marreiros, M. G. Finn, David S. Sholl, Ryan P. Lively

**Affiliations:** † School of Chemical and Biomolecular Engineering, Georgia Institute of Technology, Atlanta, Georgia 30332-0100, United States; ‡ Crystallography Lab, Emory University, Atlanta, Georgia 30322, United States; § School of Chemistry and Biochemistry, Georgia Institute of Technology, Atlanta, Georgia 30332-0100, United States; ∥ Oak Ridge National Laboratory, Oak Ridge, Tennessee 37830, United States

## Abstract

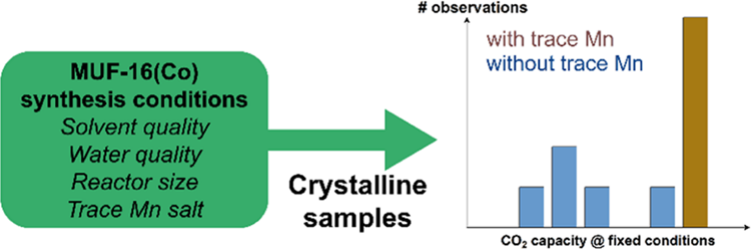

The effects of impurities on reaction precursors for metal–organic framework (MOF) synthesis have not been studied in extensive detail. The impact of these impurities can be an important factor while considering scale-up of these materials. In this work, we study the apparently positive impact of the presence of manganese ions for the synthesis of a Co-based MOF, Massey University Framework-16 (MUF-16). The presence of a trace amount of manganese in the reaction mixture led to consistently high CO_2_ uptake across multiple batches. Characterization including X-ray diffraction, scanning electron microscopy, Fourier transform infrared-attenuated total reflectance, ultraviolet–visible spectroscopy, thermogravimetric analysis, X-ray photoelectron spectroscopy, and extended X-ray absorption fine structure spectroscopy led us to hypothesize that the differences in CO_2_ adsorption among materials with differing synthesis routes arise from variations in the local environment around the cobalt metal center. Aided by density functional theory calculations, we speculate that manganese ions get inserted into the structure during crystallization and act as catalysts for ligand substitution, improving the possibility for octahedral coordination of cobalt with the ligand, thus leading to Co-based pristine structures with higher CO_2_ uptakes.

## 1 Introduction

Metal–organic frameworks (MOFs) are of interest for a wide range of applications including gas separations. The enormous number of chemically distinct MOFs that are known has created opportunities to screen large collections of materials for desirable properties. When screening of this kind or direct observation indicates that a particular MOF has interesting properties, maintaining consistency in material properties as the MOF is produced at varying scales can be challenging. Addressing this challenge is important because the aim of many research studies is to use data from samples produced in small quantities (often milligram scales) to draw conclusions about applications that would involve samples produced at kilogram or ton scales.

One difficulty with considering what effects control the outcomes of MOF synthesis is simply that the synthesis of most MOFs is reported only once in the literature. Agrawal et al. conducted a meta-analysis on the syntheses reported in the MOF literature and estimated that less than 6% of all MOFs studied have been synthesized and reported by a group of authors distinct from the original report.^1^ Even among MOFs that have been synthesized many times, there can be considerable variation in reported properties. Meta-analysis of gas adsorption isotherms in hundreds of examples where replicate measurements are available in the literature has suggested that 15–20% of reported isotherms are outliers in the sense that they are inconsistent with other reported measurements.^2−4^ Wide variations in reported surface areas have also been documented in the small number of MOFs whose synthesis has been reported tens or hundreds of times.^1^ Some of the variations in surface areas have been shown to stem from inconsistent choices in data analysis, but it is also widely believed that differences in details of synthesis methods and postsynthesis treatment systematically impact the properties of MOF crystals.^5^ Understanding the source of these variations for specific materials, especially when they result from effects that might not usually be considered during laboratory-scale synthesis, could play an important role in enabling future applications of promising materials.

While multiple MOF synthesis routes, including solvothermal, electrochemical, microwave-assisted, mechanochemical, and flow synthesis have been explored, the effects of impurities and contaminants in reaction mixtures for MOFs have only rarely been investigated to the best of our knowledge.^6−8^ The potential importance of these effects can be appreciated by related studies with inorganic nanocrystals.^9−12^ For example, the presence of iodide impurities in Hexadecyltrimethylammonium bromide (CTAB) used in the synthesis of Au nanorods has been reported to strongly affect the nanorod morphology; the absence of iodine in the reaction mixture primarily produced gold nanospheres whereas iodine contamination gave a nearly 100% yield of nanorods.^12,13^ Dialkyl impurities in trioctyl phosphine have been reported to initiate nucleation by accelerating the decomposition of metal precursors in the synthesis of lead- and cadmium-based quantum dots.^14,15^

In this paper, we investigate the surprising role of manganese ions in the synthesis of a cobalt-based MOF, Massey University Framework-16 (MUF-16).^16−18^ MUF-16 shows excellent selectivity for carbon dioxide over hydrocarbons and nitrogen and thus has the potential to be a useful material for industrial gas separations including postcombustion capture, natural and biogas upgrading, and purification of hydrocarbons.^19−23^ Its high CO_2_ uptake capacity (∼2 mmol/g at 100 kPa) and moderate heat of adsorption for CO_2_ (∼32 kJ/mol at low uptakes) imply low energy requirements for regeneration, making it an attractive material for large-scale separations.^18^ MUF-16 exhibits a somewhat rare preference for CO_2_ over heavier C_2_ and C_3_ hydrocarbons, making it an interesting candidate for the purification of mixtures of these hydrocarbons.^24^

The solvothermal synthesis of MUF-16(Co) established by Qazvini et al. involves heating cobalt acetate tetrahydrate, 5-aminoisophthalic acid, methanol, and water at 85 °C under autogenous pressure for 2 h, generating pink (or purple) crystals.^18^ In performing a series of repeated syntheses of MUF-16(Co) we observed considerable variation in CO_2_ uptake capacities among different batches (more details are given below). This prompted us to explore the source of these variations. As we show in this article, we found evidence that the presence of manganese ions (Mn^2+^ or Mn^3+^) above a threshold concentration reproducibly leads to the highest CO_2_ adsorption capacity in this MOF. Our results suggest that manganese ions at the relevant concentration are often present in practice under the synthesis conditions outlined above, even though manganese does not appear in the nominal list of elements included in the synthesis. By using multiple repeat syntheses and a broad suite of characterization techniques, we hypothesize that low-performing batches with a lower level of CO_2_ adsorption suffer from pore collapse or dangling linker defects that cause lower porosity and hence lower levels of adsorption.

We performed a variety of control experiments to probe the unexpected effect of manganese on the properties of MUF-16(Co). Briefly, we performed repeat synthesis of the MOF in the absence of manganese using 45 and 200 mL reactors, DI water, and ACS-grade MeOH (these experiments are denoted Series R below), obtaining a broad distribution of observed CO_2_ loadings. To test the effect of solvent grade, the synthesis was performed using combinations of varying grades of MeOH (ACS, HPLC) and water (DI, HPLC); these experiments are denoted Series S. We also performed synthesis using tap water to deliberately include ppm levels of ions in water (denoted Series T). Information on water purity from the City of Atlanta Department of Watershed Management first suggested to us that manganese might be playing a role in the MOF’s synthesis.^25^ We then tested multiple concentrations of Mn in the reaction mixture (denoted as Series M*x*_*y*). Finally, we tested the possibility of deliberately inserting manganese in the MOF’s structure using DFT calculations and ICP-OES to conclude that Mn gets inserted in the structure.

## 2 Methods

### 2.1 Materials and Reactors

5-Aminoisophthalic acid (Thermo Scientific, 98%), cobalt(II) acetate tetrahydrate (Alfa Aesar, 98.0–102.0%), manganese(II) acetate (Sigma Aldrich, 98%), manganese(III) acetate dihydrate (Sigma Aldrich, 97%), methanol (ACS grade, ≥99.8%, and HPLC grade, ≥99.9%, as specified), and water (HPLC grade, ≥99.9%) were used without further purification. DI water was generated using an Elga DV35 LabWater system. 200 mL (part 4748) and 45 mL (part 4744) reactor vessels were purchased from Parr Instrument Company, USA.

### 2.2 Synthesis

The synthesis procedure reported by Qazvini et al. was followed with changes noted below as appropriate.^18^ The procedure consisted of mixing cobalt(II) acetate tetrahydrate (0.2083 g, 0.84 mmol), 5-aminoisophthalic acid (0.6 g, 3.33 mmol) with methanol (26.67 mL) and water (1.67 mL) in a 45 mL Teflon-lined acid digestion pressure vessel. The mixture was ultrasonicated (Branson 450 digital sonifier, USA) for 20 min before being allowed to react at 85 °C for 2 h in a preheated oven (Forced Air Oven, 2.3 cubic feet, VWR, USA) and then cooled back to room temperature. The crystals were then washed at least three times by soaking in 35 mL or more MeOH with each wash being 12 h or longer. The resulting powders were dried under vacuum at 150 °C overnight. For all syntheses in 200 mL vessels, the amount of each reagent was four times greater than that of the 45 mL reactors. The nomenclature of the syntheses reported in this work is described below:

- R1–R4: This series was synthesized in 45 mL liners exactly as mentioned above, except for R4 for which a 200 mL Teflon liner was used instead. DI water and ACS-grade MeOH (Sigma Aldrich) were used. The electrical resistivity of DI water used was 15 MΩ cm.
- Tap water control group T1, T2: These syntheses used tap water instead of DI water. T1 was synthesized in a 45 mL reaction vessel and T2 in a 200 mL vessel. The electrical resistivity of the tap water used was <10 MΩ cm.
- Test group for manganese, M*x*_*y* (*x* = oxidation number of manganese, here 2 or 3): Mn^x+^ (Mn(II) acetate or Mn(III) acetate dihydrate) was added to the reaction mixture with concentration of *y* millimoles Mn mol^–1^ Co. HPLC grade solvents were used to ensure consistency in solvent quality for this series.
- Solvent grade test: S1–S3: In S1 both the water and MeOH used were HPLC grade. In series S2 HPLC grade water and ACS-grade MeOH were used. Repeat syntheses of S2(I) and S2(II) were performed with the same protocol. In Series S3 the solvents were DI water and HPLC grade MeOH.
- Manganese insertion in the MOF: Further experiments were performed with synthesis mixtures containing only Mn^2+^ (i.e., only Mn(II) acetate, no cobalt(II) acetate tetrahydrate) to form MUF-16(Mn) and a batch containing 1:1 molar ratio of Mn(II) acetate and cobalt(II) acetate tetrahydrate (0.42 mmol each) denoted MUF-16(Co/Mn).

The samples are activated under vacuum at 130 °C.

### 2.3 Powder X-ray Diffraction (PXRD) and Single-Crystal X-ray Diffraction (SCXRD)

PXRD patterns were recorded for all samples postactivation using a Rigaku Miniflex Powder XRD to evaluate long-range crystallinity and establish crystallographic phase(s). Cu Kα X-rays (λ = 1.5406 Å) were used, and 2θ was varied between 4 and 60° at 10°/min and a step size of 0.01°.

The crystal structures of various samples were resolved from single crystals of batches S2(II) and R3 by using a Rigaku XtaLAB diffractometer with Cu Kα X-rays. The data was integrated to get structure cif files using the CryAlisPro package.

### 2.4 Scanning Electron Microscopy (SEM)

Crystal samples were examined by using SEM with a Hitachi 8320 field emission scanning electron microscope. Samples were sputtered with Au–Pd using a Quorum Q-150 V ES Plus.

### 2.5 Single Component Gas Isotherms

Single component CO_2_, N_2_, and CH_4_ isotherms were measured at 30°C using a commercial adsorption analyzer, Micromeritics ASAP (Accelerated Surface Area and Porosity) 2020 Plus. At least 60 mg of the sample was activated at 150 °C for at least 12 h under vacuum (<10 μmHg pressure) prior to measurement. The dead volume in the sample tube was measured using helium gas. Each pressure point measurement was allowed to equilibrate until the rate of change of pressure in an equilibration interval was less than 0.01% of the average pressure during the interval. The equilibration interval was set at 60 s for pressure <35 mmHg and 35 s for higher pressures. The minimum possible equilibration time for each pressure point was set to 10 times the equilibration interval.

### 2.6 Thermogravimetric Analysis

Crystal samples were measured for thermal stability on a TA Q5000 Thermogravimetric Analyzer. The temperatures were ramped up to 150 °C, held there for at least 120 min under a nitrogen flow, and then ramped up to 700 °C at a rate of 5 °C/min under air before cooling back down.

### 2.7 Attenuated Total Reflectance-Fourier Transform Infrared (ATR-FTIR) Spectroscopy

*Ex situ* FTIR spectra were recorded using a Thermo Scientific Nicolet iS50 FTIR equipped with an iS50 ATR module between wavelengths of 600 and 4000 cm^–1^. Background spectrum was measured using dry KBr and was subtracted from all reported spectra.

### 2.8 X-ray Photoelectron Spectroscopy (XPS)

X-ray photoelectron spectra were acquired on a Thermo Fisher Scientific K-Alpha spectrometer. The resulting data were used to compare Co oxidation states in samples.

### 2.9 UV–Vis Spectroscopy

UV–visible absorbance spectra were measured for samples digested in nitric acid with a Varian Cary 5000 UV–visible/NIR spectrophotometer, using a 1 mm-path length quartz cuvette, at a scan rate of 600 nm min^–1^. In this instrument, the samples were illuminated by a tungsten halogen and a deuterium arc light source. The spectral bandwidth across the UV–visible region is 0.01–5.00 nm. One mg of each MOF sample tested was dissolved separately in 13 mL of 1 M nitric acid solution (aqueous). The linker was dissolved in 10 mL of aq. nitric acid solution. Successive 5-fold dilutions of each individual stock solution were performed twice using the same 1 M nitric acid solvent. All of the measurements were carried out at room temperature.

Diffuse reflectance UV–Vis spectra were also measured for four samples in the powder state on a Varian Cary 5000 spectrophotometer equipped with a Varian Internal Diffuse Reflectance Accessory 2500. Air was used as the reference material. The spectra were recorded on dry solid samples in a wavelength range of 200–800 nm at a scan rate of 600 nm min^–1^. All the measurements were carried out at room temperature. The reported reflectance percentages were normalized to have 100% reflectance at 800 nm.

### 2.10 Extended X-ray Absorption Fine Structure (EXAFS) Spectroscopy

An EXAFS experiment was conducted using an EasyXAFS300+ to probe the local environment of the Co metal center in the MOF. X-ray adsorption around the energy of Cobalt’s K edge at 7709 eV was accessed using a Si 771 monochromator. Data processing was done using the open-source software Athena. The data were used to calculate absorption coefficients μ(*E*) given by
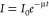
1where *I*_0_ and *I* are incident and transmitted intensities of X-rays, respectively, which are measured during the experiment. The EXAFS oscillations are defined as
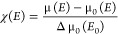
2

χ(*k*) is then obtained using . χ(*k*) is further Fourier transformed to give χ(*R*).

### 2.11 Inductively Coupled Plasma Optical Emission Spectroscopy (ICP-OES)

The final concentration of metals, Mn and Co, in sample M2_4.9 was obtained by conducting ICP-OS on a Thermo Fisher iCAP Pro XP Duo located at Intertek Chemical and Pharmaceutical lab, Whitehouse, NJ, USA. Two mL of an acid mixture (H_2_SO_4_/HNO_3_/HClO_4_ = 3:1:1) was added to 35 mg of sample in a 100 mL Gerhardt digestion vessel. The sample was digested via reflux in which the temperature of the vessel was ramped up to 240 °C and held constant for 15 min. It was then cooled and further diluted with DI water to get 10 mL of final volume of the analyte. This solution was filtered before analysis.

The instrument was calibrated using NIST traceable standards, and a two-point calibration curve was constructed. Calibration was confirmed by reading a calibration check standard prepared independent of the calibration standards. The prepared sample solution was then analyzed, and the measured emission or absorbance was compared with the calibration curve to determine the concentration in the prepared sample solution. The concentration of cobalt was determined using a wavelength of 230.786 nm (Axial) and Mn was determined using 257.610 nm (Axial).

### 2.12 Energy of Mixing Calculations Using Density Functional Theory (DFT)

The possibility of insertion of Mn in the backbone of the MOF was estimated with DFT calculations by calculating the energy of mixing of metal centers as described by Ibikunle et al.^26^ Ground state energy calculations and structure optimizations were performed using the Vienna Ab initio Simulation Package (VASP).^27−29^ Plane wave basis sets and projector augmented wave (PAW) pseudopotentials along with the Perdew, Burke, and Ernzerhof (PBE) generalized gradient approximation exchange correlational function were used.^30,31^ Γ point sampling was used with a plane wave energy cut off of 520 eV and D3 dispersion corrections using the Becke–Johnson damping function.^32^ The CIF structure as reported by Qazvini et al. was optimized prior to use for this analysis in a calculation that allowed for atom positions and the unit cell volume to relax. After this, some Co metal centers were substituted with Mn to obtain heterometallic structures with varying Mn metal mole fractions. For calculating energies for these structures, all atoms were allowed to relax while the unit cell dimensions were kept fixed. The energy of mixing is defined as
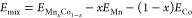
3where *x* is the metal mole fraction of Mn in the bimetallic material, *E*_Mn*_x_* Co_1–*x*__ is the energy of the bimetallic unit cell and *E*_Co_ and *E*_Mn_ are energies of homometallic structures with Co and Mn, respectively.^26^ The quantity only accounts for enthalpy and does not include entropic contributions, which always favor disordered systems relative to ordered systems.

## 3 Results and Discussion

**Figure 1:**
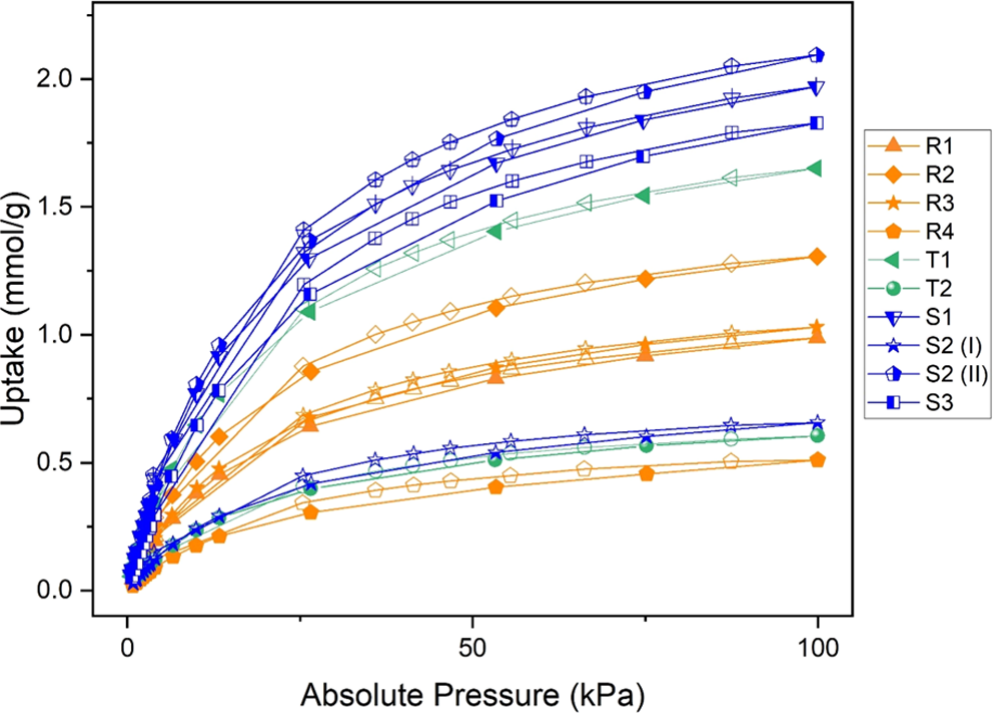
Single component CO_2_ isotherms at 30 °C for repeat batches for MUF-16 (Co) synthesis without Mn ions. Series R: repeat synthesis. Series S: solvent grade testing, and Series T: with tap water. Open symbols show uptake values during desorption.

**Figure 2:**
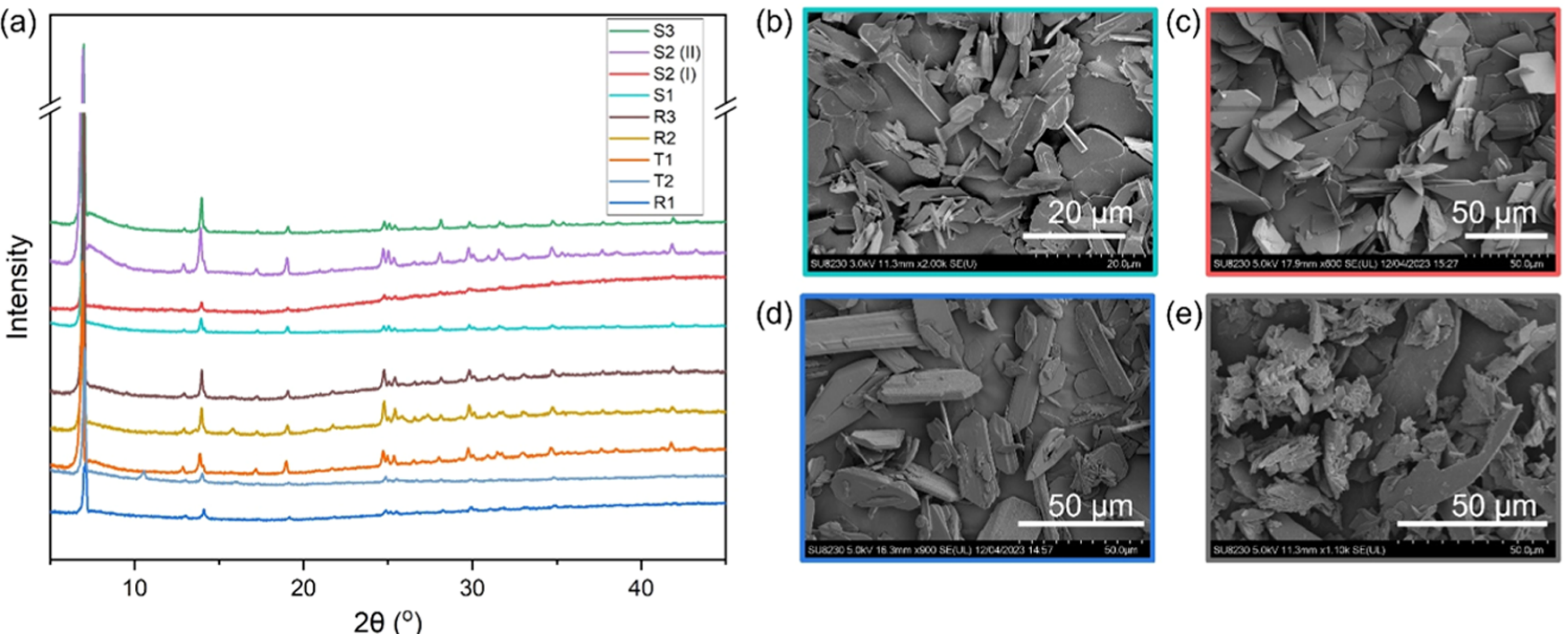
(a) PXRD patterns for R, S, and T series synthesis. The patterns follow the order noted in the legend. SEM images for batches (b) R3, (c) S1, (d) T2, and (e) S2(I).

We first consider the properties of MUF-16(Co) when DI water is used in the synthesis (Series R). Figure 1 shows the CO_2_ adsorption isotherms for the samples from Series R. These Series R samples all show similar PXRD patterns (Figure 2a) that are in good agreement with the data reported by Qazvini et al.^18^ N_2_ physisorption in this MOF does not happen at a sufficient rate at cryogenic conditions (77 K) to enable reliable surface area measurements, thus we turned to CO_2_ adsorption isotherms as a performance metric to compare sample quality. It is clear from Figure 1 that the CO_2_ loading varies strongly from batch to batch, and all of the samples show lower CO_2_ loadings than the experiments of Qazvini et al., which reported a capacity of ∼2 mmol/g at 100 kPa. At this pressure, the CO_2_ loading of batch R4 was only 0.51 mmol/g, for instance. SEM images from Series R (batch R3) in Figure 2b show a mixture of needle- and sheet-like structures that appear crystalline. The sizes vary over a broad range but there is preferential growth in one or two directions giving the crystals a sheet- or needle-like appearance.

Because none of the syntheses in Series R gave results for CO_2_ adsorption in agreement with earlier reports, we examined the potential impacts of solvent quality in Series S. A striking observation from Figure 1 is that three of these samples, S1, S2(II), and S3, give CO_2_ isotherms that are similar to the report of Qazvini et al., with S2(II) giving a CO_2_ loading of 2.09 mmol/g at 100 kPa. One of these samples, S2(I), synthesized with ACS-grade MeOH and HPLC grade water, gave very low CO_2_ adsorption. Similar to the materials in Series R, these crystals also have PXRD patterns that are consistent with earlier reports and the expected crystal structure, as shown in Figure 2a. SCXRD analysis for batches R3 (CO_2_ uptake of 1.03 mmol/g at 100 kPa and 30 °C) and S2(II) (CO_2_ uptake of 2.09 mmol/g at 100 kPa and 30 °C) resulted in similar structures as shown in Figure S3 and Table S2, and these structures are very similar to the results reported by Qazvini et al. (Table S2).^18^ However, the SEM images for materials from series S are different from Series R in that they show a significantly higher percentage of sheet-like structures with more consistent size distribution. This is a consistent observation in all high-performing samples (Figure 2(c,e)).

To further probe the role of solvent quality, we performed two additional syntheses using tap water instead of DI water (denoted series T). One of these samples, T1, showed higher amounts of CO_2_ adsorption than all of the Series R materials, while sample T2 showed a relatively low capacity for CO_2_ (see Figure 1). These materials also showed PXRD patterns consistent with those of the materials discussed above (Figure 2). SEM images also show crystals consistent with the previously stated observations that low-performing samples (T2, Figure 2d) show a mixture of sheet- and needle-like structures, whereas T1 (Figure S2(a)) shows crystal morphology similar to batch S1. The strong variations in performance among the set of MUF-16 (Co) samples shown in Figure 1 suggested that it would be useful to more systematically understand how to reduce this variability.

**Figure 3:**
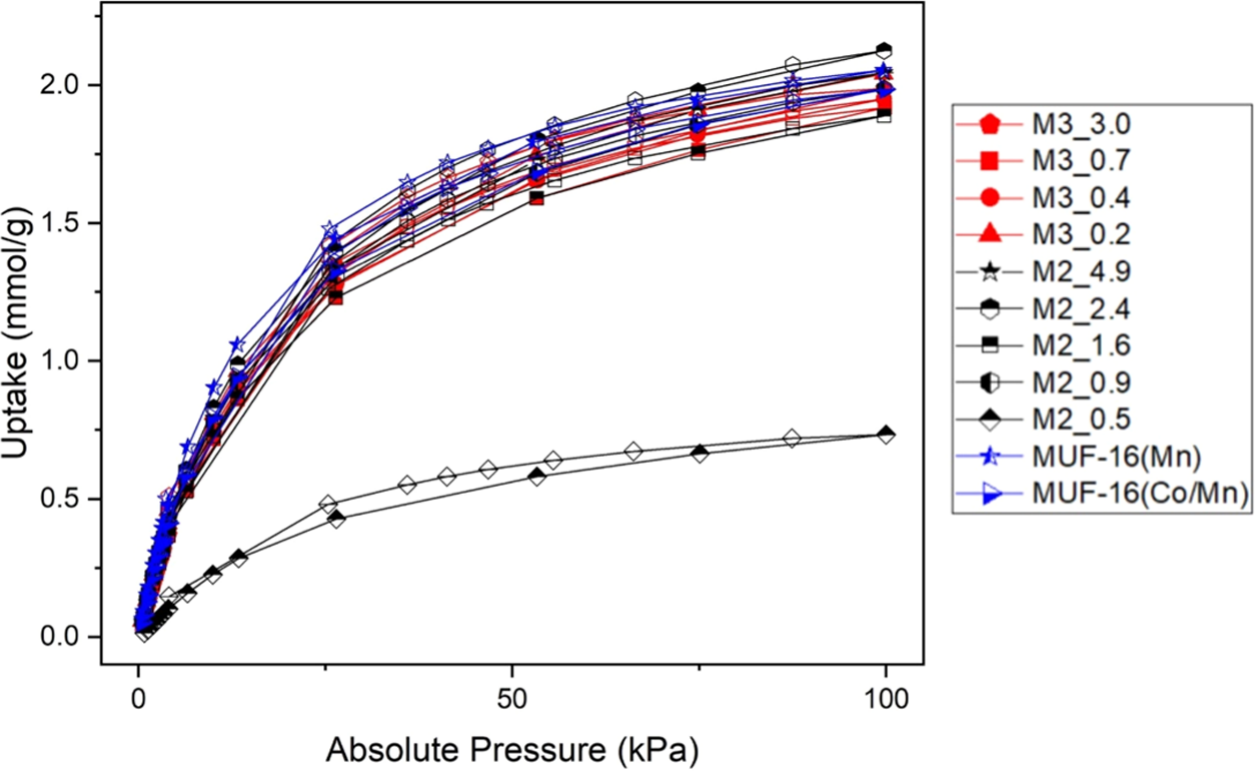
Single component CO_2_ isotherms at 30 °C for syntheses with the addition of Mn ions where M*x*_*y* indicates addition of Mn^*x*+^ ions at a concentration of *y* mmol Mn^*x*+^/mol Co^2+^. MUF-16(Co/Mn) was synthesized with 1:1 molar concentration of Co^2+^ and Mn^2+^ in the reaction mixture. Open symbols show uptake values during desorption.

Examination of water quality information from the City of Atlanta (where our laboratory is located) led us to hypothesize that small concentrations of manganese in the MUF-16 (Co) synthesis solution could have significant impacts on the properties of the resulting MOF. To test this concept, we performed a number of syntheses with controlled quantities of Mn. Since Co(oAc)_2_ was used as a metal precursor for cobalt, acetate salts were chosen as Mn sources to rule out any anion effects. Our data from the experiments denoted Series M*x*_*y* indicate the addition of Mn^*x*+^ ions in the synthesis solution at a concentration of y mmol Mn^*x*+^/mol Co^2+^. PXRD and SEM results from these samples are shown in Figures S1(a) and S2, showing agreement with the general observations described above; the PXRD patterns are consistent with each other and with samples for Series R, S, and T and SEM images show sheet-like structures for all high-performing M*x*_*y* batches synthesized (Figure S2). CO_2_ adsorption isotherms for the materials in Series M*x*_*y* are shown in Figure 3. A striking feature of these measurements is that all batches with synthesis solutions containing Mn^2+^ or Mn^3+^ concentrations in excess of 0.9 mmol of Mn^*x*+^/mol of Co^2+^ resulted in CO_2_ adsorption that are consistent (within experimental uncertainty) with the original data of Qazvini et al. and the material with the highest CO_2_ loading shown in Figure 1. We do see some hysteresis of the order of 0.05 mmol/g during desorption in some batches. We attribute this to the variation to error of pressure transducers on the ASAP 2020.

N_2_ and CH_4_ isotherms for batches M2_4.9 and M3_3.0 are given in Supporting Information Section 1. They show minimal CH_4_ and N_2_ uptake and the data are comparable to the data reported by Qazvini et al., which implies that the use of manganese has no adverse effect on the selectivity of MUF-16 for CO_2_ over N_2_ or CH_4_. All isotherm data shown in Figures 1 and 3 are tabulated in the Supporting Files.

**Figure 4:**
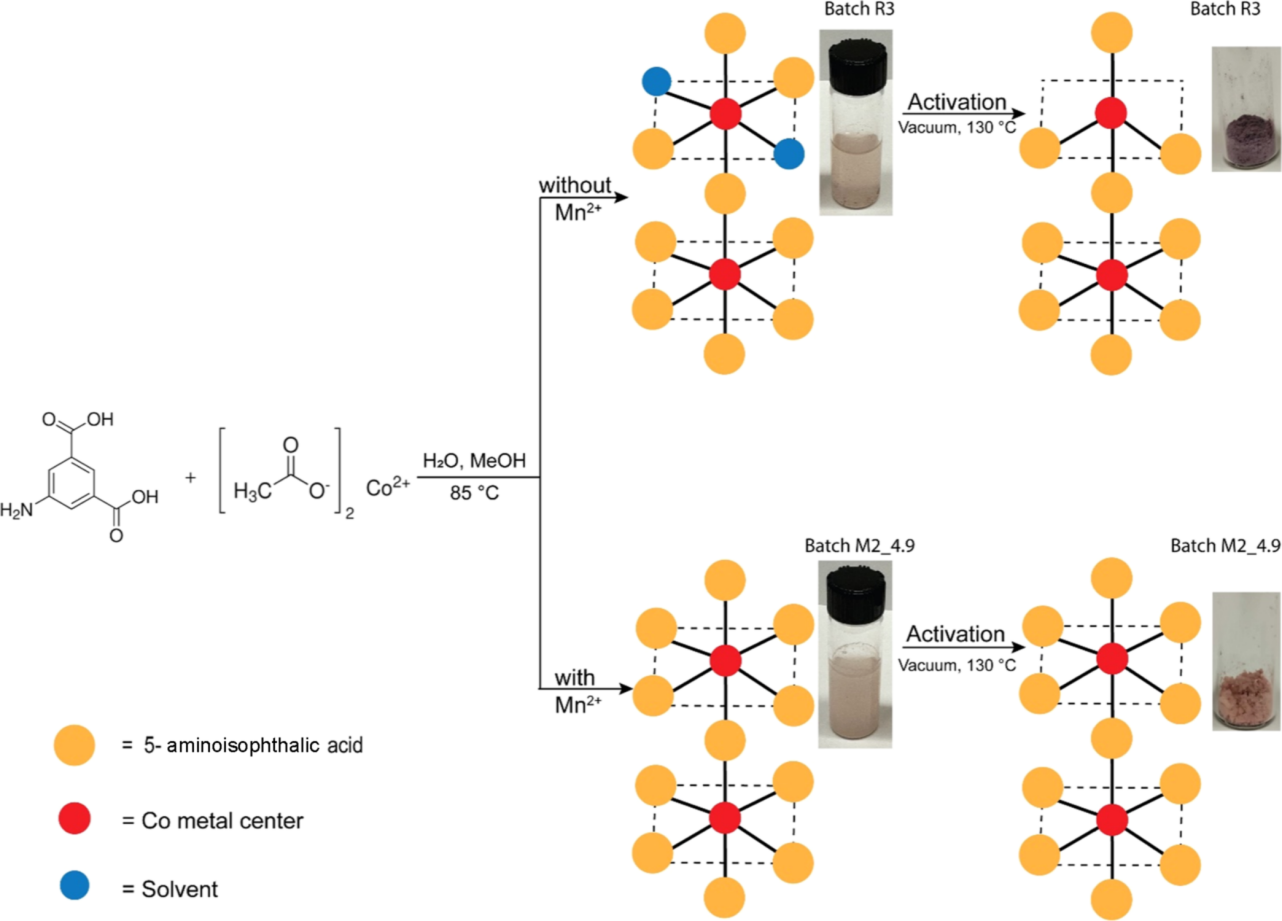
Proposed hypothesis for the effect of Mn ions on the formation of MUF-16. (Top) MOFs with low CO_2_ uptake include metal centers that are surrounded or partially surrounded by solvent molecules in the solvated state, which leave upon activation, thus leading to pore collapse or dangling linker defects. (Bottom panel) High-performing MOFs have metal centers that stay coordinated to linkers octahedrally postactivation. Images in the inset figure show that in solvated state preactivation, both high-performing batch M2_4.9 and low-performing batch R3 are pink in color. After heating under vacuum, the low-performing batches turn purple, consistent with the geometry of the cobalt ion.

The discussion above grouped our samples into examples where the CO_2_ adsorption is large and consistent with earlier reports (M*x*_*y* except M2_0.5, S1, S2(II) and S3), and examples that gave much lower amounts of CO_2_ adsorption (series R, series T, and S2(I)). We reiterate that all of these samples showed good crystallinity as judged by PXRD. All of these samples were pink in the solvated state, but samples with high levels of CO_2_ adsorption retained this color after activation while the other samples turned purple after activation (Figure 4). This is a useful observation since Co octahedral coordination complexes with weak field ligands are often pink (as in the case of Co(oAc)_2_·4H_2_O, [Co(H_2_O)_6_]^2+^) and tetrahedral complexes are often blue (for example [CoCl_4_]^−^) based on crystal field theory for coordination complexes.^33,34^ The observed color change suggests that in the high CO_2_ adsorption samples all or nearly all of the Co centers are octahedral as expected from the MOF’s crystal structure, but in the samples with lower CO_2_ adsorption a fraction of the Co centers is coordinated to solvent species before activation and convert to tetrahedrally coordinated centers upon activation (Figure 4). We hypothesize that this leads to pore collapse in those tetrahedral Co-containing unit cells and thus causes low CO_2_ uptake. This also offers an explanation as to why there is a broad range of performance observed among the low-performing batches, as the percentage of collapsed pores would be proportional to tetrahedral metal centers.

**Figure 5:**
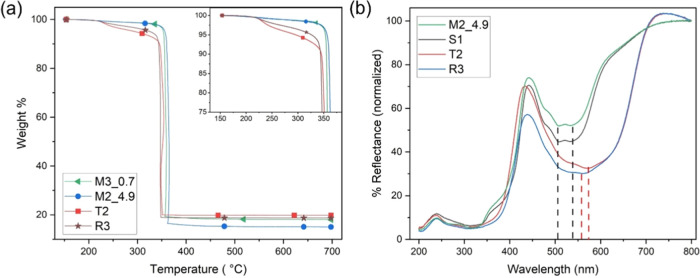
(a) TGA profiles for MUF-16, R3, T2, M2_4.9, and M3_0.7. The weight percentage is calculated based on dry weight after activating *in situ* at 150 °C for 2 h. Inset shows the TGA profile for the samples between temperatures of 150 and 380 °C. Data was collected at temperature intervals smaller than 1 °C; symbols on the curves are to guide the eye. (b) Diffuse reflectance UV–vis spectra for samples M2_4.9, S1, T2, and R3. The data have been normalized using % reflectance at 800 nm. Black and red dashed lines indicate major absorbance peaks for high and low-performance batches, respectively.

Further evidence for the differences in metal coordination states between the samples with high and low CO_2_ adsorption comes from thermogravimetric analysis (TGA). TGA was performed by heating samples in air to quantify the metal composition among different batches, as shown in Figure 5a. The samples were activated in situ under nitrogen at 150 °C for at least 2 h prior to heating in air. The batches with lower CO_2_ uptakes (R3 and T2) show a gradual decrease in residual weight from 200 to 350 °C, at which point the sample mass undergoes a sharp decline. This is not the case, however, for high-performing syntheses (M3_0.7 and M2_4.9) where the MOF weight remains constant up to approximately 350 °C and then sees a sharp decline at higher temperatures. TGA curves from other batches also align with this observation (Figure S5). The lower apparent decomposition temperature of the non-Mn samples suggests the presence of loosely bound species in the material, which we speculate are dangling linkers (i.e., linkers that are only partially attached to a metal center). These dangling linkers likely exist because some of the Co metal centers are coordinated to solvent molecules. X-ray photoelectron spectroscopy (Figure S6) for the Co 2p region showed that samples with low CO_2_ uptake show a dominant shoulder at around 786 eV, which corresponds to a Co 2p_3/2_ satellite feature in the Co(II) oxidation state spectrum. This shoulder is less dominant in high-performing batches.^35^ This indicates that Co environments and as a result, oxidation states in the low-performing batches differ from the high-performing ones; however, the difference is small and could also potentially be attributed to experimental noise.

The local environment of the cobalt metal center in the MOFs was investigated in the dissolved state using UV–vis spectroscopy (Figure S9). The UV–vis results do not show any significant differences between the batches tested, indicating that in the solvated state, cobalt exists in the same coordination state irrespective of uptake performance of the MOF. This observation aligns with the behavior with respect to the color shown by these samples stated above, supporting the idea that in the solvated state (i.e., before activation) all the samples have similar octahedral Co coordination. Diffuse reflectance UV–vis spectra that were measured for powder samples in the solid state clearly shows a difference in Co metal center coordination. The normalized reflectance spectra are shown in Figure 5b, and the major absorbance bands are marked by dotted lines. High-performing batches (M2_4.9 and S1) see a major absorbance contribution in the 500–540 nm band, whereas for low CO_2_ uptake batches (T2 and R3), the bands shift to 550–575 nm. The absorbance band in the case of T2 and R3 is also broader and more intense than the high-performing batches. This shift and broadening of absorbance peak indicate that the Co coordination for at least some metal centers in low-performing batches is tetrahedral. In a study on Co(II) exchanged zeolite X, Sebastian et al. also reported a change in the observed color of batches from pink to purple when the Co coordination state changed from octahedral to tetrahedral, based on comparable trends in DR UV–Vis spectra.^36^

**Figure 6:**
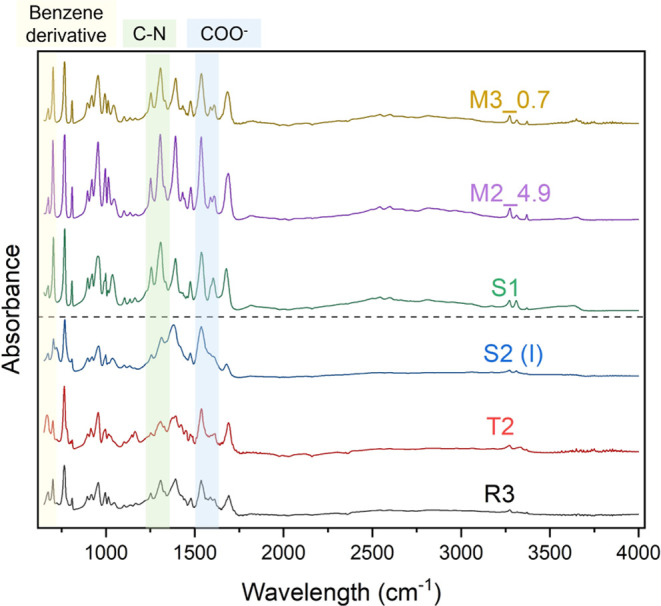
ATR-FTIR spectra for batches M3_0.7, M2_4.9, S1 with >1.9 mmol CO_2_/g MUF-16 uptake at 100 kPa (top panel), and batches S2(I), T2, R3 with 0.66, 0.6, and 1.03 mmol/g uptake at 100 kPa, respectively (bottom panel).

We sought to understand the effect of dangling linkers and varying cobalt coordination environments on various chemical functionalities present in the MOF. Figure 6 shows FTIR-ATR spectra for six samples, with benzene derivative frequencies (in yellow) between 700 and 720 cm^–1^, strong C–N stretching for an aromatic primary amine (in green) between 1237 and 1347 cm^–1^ and COO– frequencies (in blue) between 1486 and 1570 cm^–1^. While all of the observed bands in the spectrum were present in all samples tested, samples with low CO_2_ uptakes (S2(I), T2, R2) show greater broadening for most bands compared to the more well-defined bands in the samples with high CO_2_ uptake. The peak broadening seen in the former samples is often indicative of mechanical strain or disorder.^37,38^ The dangling linker defects discussed above are one plausible source of this kind of disorder in poorly performing batches. It is worth noting that no additional dominant vibrations appear in the spectra for the poor-performing materials, meaning that there are no obvious side reaction products. FTIR-ATR spectra for the other samples reported are given in Figure S8.

**Figure 7:**
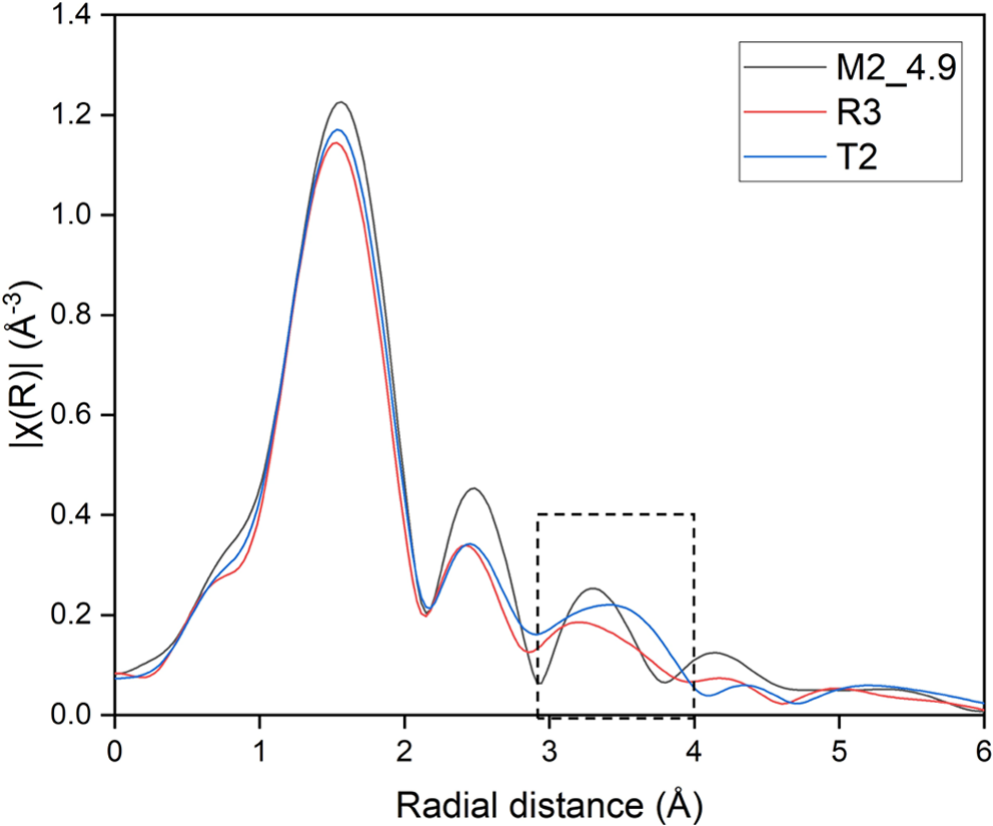
EXAFS spectroscopy for batches M2_4.9, R3, and T2. χ(*R*) values show coordination shells for the cobalt metal center. The dashed box highlights the 3rd coordination shell.

To probe the local environment of cobalt in the MOF in the solid state, EXAFS spectroscopy was used to obtain X-ray absorption. Figure 7 shows χ(*R*) for batches M2_4.9, R3, and T2. There are significant differences in the third coordination shell of cobalt among the various samples, where samples R3 and T2 show nonzero values of |χ(*R*)| over a broader range as compared to the manganese-containing the M2_4.9 sample, indicating a wider range of bond lengths in the distance between 3 and 4 Å for the former samples. This further supports the hypothesis that the local cobalt environments in these samples are different in the solid state, which potentially leads to differences in the CO_2_ uptake performance. We speculate that dangling linkers would broaden the distribution of apparent bond lengths around the cobalt metal centers, as observed in the third and fourth coordination shells for samples R3 and T2. In principle, FEFF calculations could be carried out to compare the obtained spectra with a simulated pattern; however, the use of a bench-scale equipment to carry out these experiments limits the resolution of the acquired data. Therefore, we restrict this analysis to a qualitative comparison between the samples tested.

**Figure 8:**
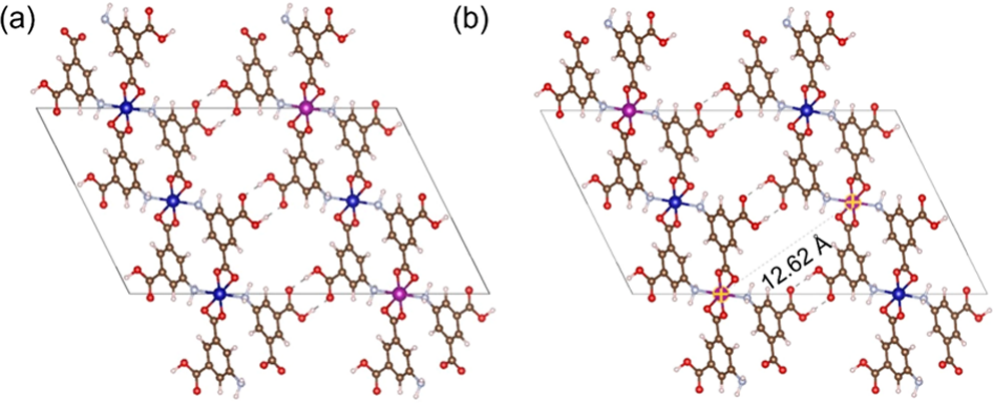
DFT optimized structures containing (a) Co_3_Mn_1_ and (b) Co_2_Mn_2_. In the latter structure, the Mn–Mn distance is 12.6 Å. The remaining structures are given in the Supporting Information. Blue = Co, pink = Mn, brown = carbon, red = oxygen, faint blue = nitrogen, and white = hydrogen.

We showed in Figure 3 that including small quantities of Mn in the synthesis solution yielded MOF samples with reproducible CO_2_ isotherms. Although the relative amounts of Mn and Co in the synthesis solution are well-defined, it is more challenging to measure whether Mn is incorporated into the MOF framework. We attempted to quantify the presence of Mn using XPS but could not detect Mn. XPS also did not detect the presence of manganese in any of M3 and M2 series synthesis (Figure S6). If present, the concentrations of Mn are below the detectable limits in these cases. The possibility of insertion of manganese in the backbone of the MOF was probed using DFT calculations. We assessed the energetic driving force for Mn to substitute Co in the structure by calculating energies for mixing as described in eq 3. Figure 8a,b shows the structures considered with 25 and 50% Mn composition. Other structures are given in Figure S10 in the Supporting Information. For all of the compositions and structures tested, the energy of mixing is slightly less than 0, indicating that there is some thermodynamic driving force for Mn insertion over Co, even though the magnitude of energy is small (Table 1). These calculations cannot provide information on any kinetic effects associated with crystallization of the MOF, but they suggest that when both Mn and Co are present in the synthesis solution it is reasonable to expect both metals to be incorporated into the MOF. This means that in all of the batches reported in Figure 3, there is a high probability of the presence of Mn in the framework; however, the ratio of Co and Mn in the framework is expected to be of the order of 1000:1. This hypothesis was tested for batch M2_4.9 using ICP-OES. We obtained a concentration of 11.9% by weight for Co and 399 ppm (μg/g) for Mn resulting in an atomic ratio of 3.6 × 10^–3^ Mn per Co. The resulting ratio is in line with the concentration ratio in the synthesis mixture, which, as indicated by the naming convention used, is 4.9 × 10^–3^ Mn per Co.

**Table 1:** Energies of Mixing Corresponding to the Five Structures Tested Obtained from DFT Calculations

arrangement	*E*_mix_ (kJ/mol) per unit cell
Co_3_Mn_1_	–0.21
Co_2_Mn_2_ (Mn–Mn distance = 13.25 Å)	–0.02
Co_2_Mn_2_ (Mn–Mn distance = 12.62 Å)	–0.51
Co_2_Mn_2_(Mn–Mn distance = 7.87 Å)	–0.26
Co_1_Mn_3_	–0.19

Our conclusions about the inclusion of Mn in the materials were also experimentally tested using two control experiments in which MUF-16 was synthesized with the Mn salt only and Mn/Co in a 1:1 ratio using the same experimental conditions described above. PXRD patterns (Figure S1(b)) and XPS spectroscopy results show that Mn can be inserted into the lattice at approximately equal concentrations (Figure S6 and Table S3). CO_2_ uptakes for these controls are given in Figure 3; the CO_2_ capacity for these materials is similar to the high-performing Co-based samples.

The observations above have established that manganese can get inserted into the MOF structure under the synthesis conditions and that the presence of even trace levels of manganese substantially improves the reproducibility of gas adsorption properties in the MOF. It is of course interesting to speculate on the reaction mechanism by which Mn ions reduce the possibility of dangling linker defects in MUF-16(Co). Previous studies of ligand substitution reactions hint that Mn may act as a catalyst for ligand substitution during the formation of the MOF.^39^ A schematic description of one potential process of this type is shown in the SI (Section S8).

## 4 Conclusions

In this work, we investigated the effects of the presence of manganese impurities in reaction mixtures on the CO_2_ adsorption performance of MUF-16(Co). CO_2_ adsorption was used as a probe because this material shows a negligible uptake of N_2_ under cryogenic conditions. We observed that the adsorption performance of the material varied significantly over multiple batches when the MOF was synthesized in accordance with the synthesis conditions originally reported by Qazvini et al.^18^ Various control experiments led us to the conclusion that the presence of manganese in trace quantities (on the order of 1 mmol Mn/mol Co in the synthesis mixture) led to more consistent quality of crystals and larger and more consistent CO_2_ uptake. In the materials produced under these conditions, the quantity of Mn in the crystals was below detection limits in our XPS analysis, although DFT calculations suggest that some Mn may be incorporated into the crystal structure.

We performed extensive characterization on batches with low and high performance for CO_2_ adsorption. These experiments, in aggregate, lead to the hypothesis that the low-performing batches suffer from pore collapse on activation (although all samples showed similar PXRD patterns). This pore collapse is speculated to derive from the presence of residual solvent molecules bound to the metal centers instead of the linkers. This hypothesis provides a tentative explanation for the observation that we obtain a broad range of performances for gas adsorption. We hypothesize that the addition of manganese promotes the formation of the octahedral coordination geometry for cobalt by acting as a catalyst for the substitution of solvent molecules with ligands.

Our work focuses on the observation that the presence of trace amounts of impurities in the synthesis of the MOF leads to consistently better gas adsorption performance. We primarily employed bulk powder techniques to derive our conclusions about the structure at the crystal or unit cell levels. While our experimental observations support our hypothesis, the possibility of other causes for low-performance such as missing metal defects or precipitation of dense phases as a result of side reactions in the synthesis cannot be completely ruled out. We have not tested the performance of our modified synthesis against the possible presence of other impurities in the reaction precursors (e.g., other metal species), which may also have important effects at larger scales.

Our results presented here are an interesting example of a MOF synthesis in which deliberate inclusion of what might be viewed as a trace impurity in the synthesis mixture significantly improves the reproducibility of the material when its properties are characterized by gas adsorption. Advancing the application of MOFs and similar materials from initial laboratory discovery to larger scales is typically possible only when material performance is robust with respect to the inevitable variations in the purity of starting materials and other aspects of synthesis that occur at greatly differing scales of operation. The generalizability of the “impurity-driven” improvements seen in our synthesis of MUF-16(Co) to other families of MOFs is currently unclear. Nevertheless, the possibility of enhancing the quality of MOF crystals by the presence of small concentrations of substitutional metal centers is intriguing and may create opportunities for improving the synthetic reproducibility of other materials.

## Supporting Information Available

Single-crystal analysis for batches S2(II) and R3; DFT optimized structure files for energy of mixing calculations (ZIP)
SEM; PXRD; single-crystal XRD; TGA; XPS; FTIR-ATR; UV–visible spectroscopy; DFT calculations for energy of mixing; proposed mechanism (PDF)
CO_2_ isotherms experimental data (XLSX)
N_2_ and CH_4_ isotherms experimental data (XLSX)

## Notes

Notice of Copyright: This work was authored by UT-Battelle, LLC, under contract DE-AC05-00OR22725 with the US Department of Energy (DOE). The publisher acknowledges the US government license to provide public access under the DOE Public Access Plan (http://energy.gov/downloads/doe-public-access-plan).

## References

[ref1] Agrawal M.; Han R.; Herath D.; et al. Does repeat synthesis in materials chemistry obey a power law?. Proc. Natl. Acad. Sci. U.S.A. 2020, 117 (2), 877–882. 10.1073/pnas.1918484117. 31879338 PMC6969490

[ref2] Park J.; Howe J. D.; Sholl D. S. How Reproducible Are Isotherm Measurements in Metal–Organic Frameworks?. Chem. Mater. 2017, 29 (24), 10487–10495. 10.1021/acs.chemmater.7b04287.

[ref3] Bingel L. W.; Walton K. S.; Sholl D. S. Experimentally Verified Alkane Adsorption Isotherms in Nanoporous Materials from Literature Meta-Analysis. J. Chem. Eng. Data 2022, 67 (7), 1757–1764. 10.1021/acs.jced.1c00967.

[ref4] Bingel L. W.; Chen A.; Agrawal M.; et al. Experimentally Verified Alcohol Adsorption Isotherms in Nanoporous Materials from Literature Meta-Analysis. J. Chem. Eng. Data 2020, 65 (10), 4970–4979. 10.1021/acs.jced.0c00598.

[ref5] Osterrieth J. W. M.; Rampersad J.; Madden D.; et al. How Reproducible are Surface Areas Calculated from the BET Equation?. Adv. Mater. 2022, 34 (27), 2201502 10.1002/adma.202201502. 35603497

[ref6] Stock N.; Biswas S. Synthesis of Metal-Organic Frameworks (MOFs): Routes to Various MOF Topologies, Morphologies, and Composites. Chem. Rev. 2012, 112 (2), 933–969. 10.1021/cr200304e. 22098087

[ref7] Rubio-Martinez M.; Avci-Camur C.; Thornton A. W.; et al. New synthetic routes towards MOF production at scale. Chem. Soc. Rev. 2017, 46 (11), 3453–3480. 10.1039/C7CS00109F. 28530737

[ref8] Lee Y.-R.; Kim J.; Ahn W.-S. Synthesis of metal-organic frameworks: A mini review. Korean J. Chem. Eng. 2013, 30 (9), 1667–1680. 10.1007/s11814-013-0140-6.

[ref9] Liz-Marzán L. M.; Kagan C. R.; Millstone J. E. Reproducibility in Nanocrystal Synthesis? Watch Out for Impurities!. ACS Nano 2020, 14 (6), 6359–6361. 10.1021/acsnano.0c04709. 32575172

[ref10] Abutbul R. E.; Golan Y. Beneficial impurities’ in colloidal synthesis of surfactant coated inorganic nanoparticles. Nanotechnology 2021, 32 (10), 102001 10.1088/1361-6528/abc0c7. 33305737

[ref11] Lazarus L. L.; Riche C. T.; Malmstadt N.; et al. Effect of Ionic Liquid Impurities on the Synthesis of Silver Nanoparticles. Langmuir 2012, 28 (45), 15987–15993. 10.1021/la303617f. 23092200

[ref12] Smith D. K.; Korgel B. A. The Importance of the CTAB Surfactant on the Colloidal Seed-Mediated Synthesis of Gold Nanorods. Langmuir 2008, 24 (3), 644–649. 10.1021/la703625a. 18184021

[ref13] Rayavarapu R. G.; Ungureanu C.; Krystek P.; et al. Iodide Impurities in Hexadecyltrimethylammonium Bromide (CTAB) Products: Lot–Lot Variations and Influence on Gold Nanorod Synthesis. Langmuir 2010, 26 (7), 5050–5055. 10.1021/la100166f. 20205463

[ref14] Evans C. M.; Evans M. E.; Krauss T. D. Mysteries of TOPSe Revealed: Insights into Quantum Dot Nucleation. J. Am. Chem. Soc. 2010, 132 (32), 10973–10975. 10.1021/ja103805s. 20698646 PMC2924661

[ref15] Steckel J. S.; Yen B. K. H.; Oertel D. C.; et al. On the Mechanism of Lead Chalcogenide Nanocrystal Formation. J. Am. Chem. Soc. 2006, 128 (40), 13032–13033. 10.1021/ja062626g. 17017765

[ref16] Trump B. A.; Qazvini O. T.; Lee S. J.; et al. Flexing of a Metal–Organic Framework upon Hydrocarbon Adsorption: Atomic Level Insights from Neutron Scattering. Chem. Mater. 2023, 35 (3), 1387–1394. 10.1021/acs.chemmater.2c03450.

[ref17] Qazvini O. T.; Telfer S. G. MUF-16: A Robust Metal–Organic Framework for Pre- and Post-Combustion Carbon Dioxide Capture. ACS Appl. Mater. Interfaces 2021, 13 (10), 12141–12148. 10.1021/acsami.1c01156. 33661605

[ref18] Qazvini O. T.; Babarao R.; Telfer S. G. Selective capture of carbon dioxide from hydrocarbons using a metal-organic framework. Nat. Commun. 2021, 12 (1), 197 10.1038/s41467-020-20489-2. 33420024 PMC7794324

[ref19] Wang Q.; Bai J.; Lu Z.; et al. Finely tuning MOFs towards high-performance post-combustion CO_2_ capture materials. Chem. Commun. 2016, 52 (3), 443–452. 10.1039/C5CC07751F. 26512792

[ref20] Younas M.; Rezakazemi M.; Daud M.; et al. Recent progress and remaining challenges in post-combustion CO_2_ capture using metal-organic frameworks (MOFs). Prog. Energy Combust. Sci. 2020, 80, 100849 10.1016/j.pecs.2020.100849.

[ref21] Angelidaki I.; Treu L.; Tsapekos P.; et al. Biogas upgrading and utilization: Current status and perspectives. Biotechnol. Adv. 2018, 36 (2), 452–466. 10.1016/j.biotechadv.2018.01.011. 29360505

[ref22] Raganati F.; Miccio F.; Ammendola P. Adsorption of Carbon Dioxide for Post-combustion Capture: A Review. Energy Fuels 2021, 35 (16), 12845–12868. 10.1021/acs.energyfuels.1c01618.

[ref23] Sarswat A.; Sholl D. S.; Lively R. P. Achieving order of magnitude increases in CO_2_ reduction reaction efficiency by product separations and recycling. Sustainable Energy Fuels 2022, 6 (20), 4598–4604. 10.1039/D2SE01156E.

[ref24] Cai X.; Gharagheizi F.; Bingel L. W.; et al. A Collection of More than 900 Gas Mixture Adsorption Experiments in Porous Materials from Literature Meta-Analysis. Ind. Eng. Chem. Res. 2021, 60 (1), 639–651. 10.1021/acs.iecr.0c05398.

[ref25] Water Quality Reports, City of Atlanta Watershed Management. https://www.atlantawatershed.org/water-quality-reports/.

[ref26] Ibikunle I. A.; Yang Y.; Valdez N. R.; et al. Trends in Siting of Metals in Heterometallic Nd–Yb Metal–Organic Frameworks and Molecular Crystals. ACS Appl. Mater. Interfaces 2022, 14 (48), 54349–54358. 10.1021/acsami.2c15638. 36399403

[ref27] Kresse G.; Hafner J. Ab initio molecular dynamics for liquid metals. Phys. Rev. B 1993, 47 (1), 558–561. 10.1103/PhysRevB.47.558. 10004490

[ref28] Kresse G.; Furthmüller J. Efficiency of ab-initio total energy calculations for metals and semiconductors using a plane-wave basis set. Comput. Mater. Sci. 1996, 6 (1), 15–50. 10.1016/0927-0256(96)00008-0.

[ref29] Kresse G.; Furthmüller J. Efficient iterative schemes for ab initio total-energy calculations using a plane-wave basis set. Phys. Rev. B 1996, 54 (16), 11169–11186. 10.1103/PhysRevB.54.11169. 9984901

[ref30] Perdew J. P.; Burke K.; Ernzerhof M. Generalized Gradient Approximation Made Simple. Phys. Rev. Lett. 1996, 77 (18), 3865–3868. 10.1103/PhysRevLett.77.3865. 10062328

[ref31] Blöchl P. E. Projector augmented-wave method. Phys. Rev. B 1994, 50 (24), 17953–17979. 10.1103/PhysRevB.50.17953. 9976227

[ref32] Grimme S.; Ehrlich S.; Goerigk L. Effect of the damping function in dispersion corrected density functional theory. J. Comput. Chem. 2011, 32 (7), 1456–1465. 10.1002/jcc.21759. 21370243

[ref33] Equilibrium Between Two Coloured Cobalt Species, Royal Society of Chemistry; https://edu.rsc.org/experiments/the-equilibrium-between-two-coloured-cobalt-species/1.article (accessed November 2023).

[ref34] Averill B. A.; Eldredge P. General Chemistry: Principles, Patterns, and Applications; Pearson College Div, 2007.

[ref35] Cobalt X-ray Photoelectron Spectra, Cobalt Electron Configuration, and Other Elemental Information. https://www.thermofisher.com/us/en/home/materials-science/learning-center/periodic-table/transition-metal/cobalt.html.

[ref36] Sebastian J.; Jinka K. M.; Jasra R. V. Effect of alkali and alkaline earth metal ions on the catalytic epoxidation of styrene with molecular oxygen using cobalt(II)-exchanged zeolite X. J. Catal. 2006, 244 (2), 208–218. 10.1016/j.jcat.2006.09.005.

[ref37] Hadjiivanov K. I.; Panayotov D. A.; Mihaylov M. Y.; et al. Power of Infrared and Raman Spectroscopies to Characterize Metal-Organic Frameworks and Investigate Their Interaction with Guest Molecules. Chem. Rev. 2021, 121 (3), 1286–1424. 10.1021/acs.chemrev.0c00487. 33315388

[ref38] Kang X.; Lyu K.; Li L.; et al. Integration of mesopores and crystal defects in metal-organic frameworks via templated electrosynthesis. Nat. Commun. 2019, 10 (1), 4466 10.1038/s41467-019-12268-5. 31578368 PMC6775123

[ref39] Richens D. T. Ligand substitution reactions at inorganic centers. Chem. Rev. 2005, 105 (6), 1961–2002. 10.1021/cr030705u. 15941207

