# Supplementary material for: Investigating the Effect of Trace Levels of Manganese Ions During Solvothermal Synthesis of Massey University Framework-16 on CO2 Uptake Capacity
Source: Chem Mater. 2024 May 17;36(11):5378–87. doi: 10.1021/acs.chemmater.4c00137 (PMC11170933; doi:10.1021/acs.chemmater.4c00137)
Supplement: Supplementary file 2 — cm4c00137_si_002.pdf [file cm4c00137_si_002.pdf]

Supplementary Information for

Investigating the effect of trace levels of manganese ions during solvothermal synthesis of Massey University Framework-16 on CO_2 uptake capacity

Akriti Sarswat[1], John Bacsa[2], Ankana Roy[3], Joao Marreiros[1], M.G. Finn[3], David S. Sholl[1,4]*, and Ryan P. Lively[1]*

[1]School of Chemical & Biomolecular Engineering, Georgia Institute of Technology, Atlanta, GA 30332-0100, USA

[2]Crystallography Lab, Emory University, Atlanta, Georgia 30322, United States

[3]School of Chemistry & Biochemistry, Georgia Institute of Technology, Atlanta, GA 30332-0100, USA

[4]Oak Ridge National Laboratory, Oak Ridge, TN 37830, USA

*Corresponding authors

*Email(s): shollds@ornl.gov , ryan.lively@chbe.gatech.edu

Table of Contents

	Section	Page no.
1.	SEM, PXRD and single component gas isotherms	3
2.	Single crystal XRD	6
3.	Thermogravimetric analysis	7
4.	X-ray Photoelectron Spectroscopy	8
5.	FTIR-ATR	10
6.	UV-Visible Spectroscopy	11
7.	Energy of mixing calculations	12
8.	Proposed mechanism for impact on Mn on MOF crystallization	13
9.	References	14

Section 1. SEM, PXRD and single component gas isotherms

PXRD patterns for high performing batches (M3_3.0, M3_0.7, M2_4.9, M2_0.9) are given in Figure S1(a). They are in good agreement with the patterns for low performing batches shown in Figure 1.

Figure 1(b) shows PXRD patterns for batches containing high amounts of manganese. The patterns show agreement with all other PXRD patterns indicating that Mn can get inserted into the structure with similar lattice.

SEM images for batches with high uptakes (T1, M2_4.9, M3_0.7) are given in Figure S2. SEM images show that the high performing batches show sheet like structures whereas low performing batches show a mix of needle like and sheet like structures.

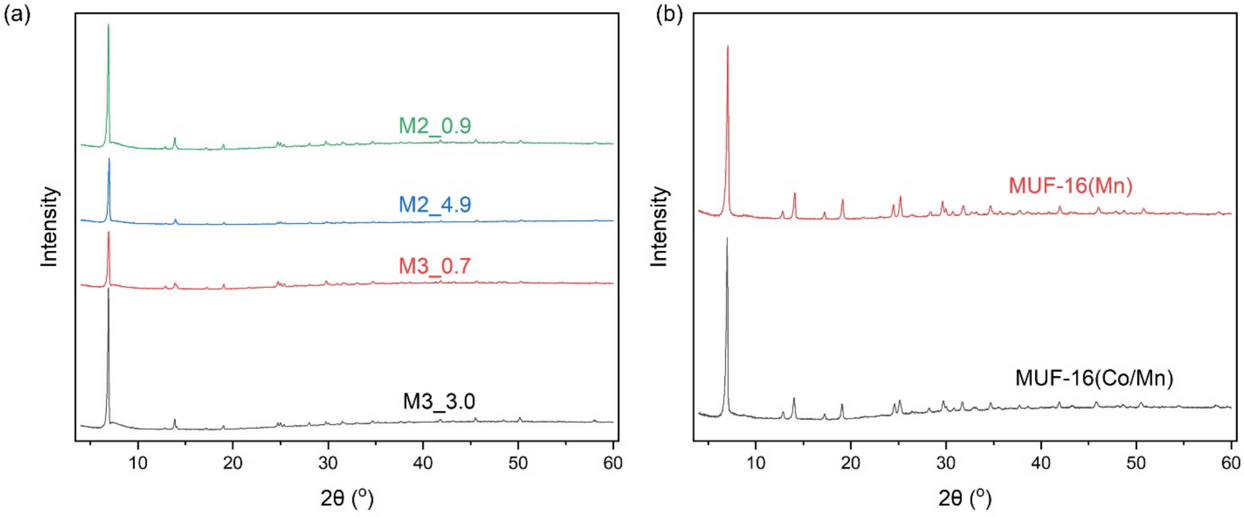

Figure S1: (a) PXRD patterns for batches containing trace amounts of manganese (b) PXRD patterns for MUF-16(Mn) synthesized with no cobalt salts and MUF-16(Co/Mn) synthesized with 1:1 molar ratio of Co and Mn in the reaction mixture.

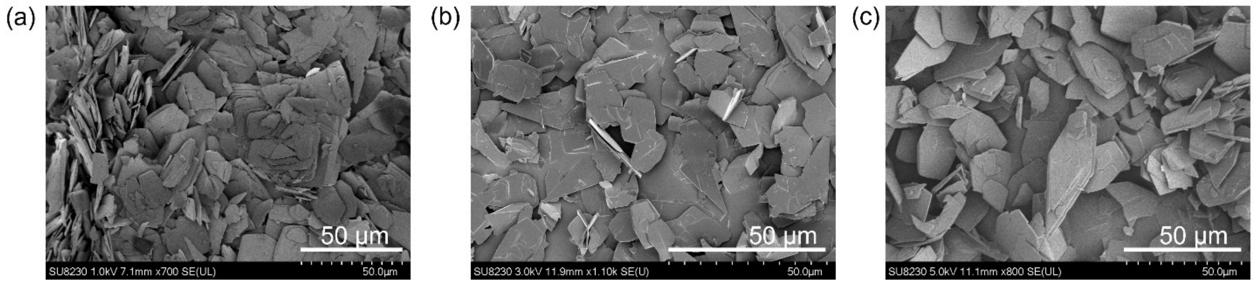

Figure S2: SEM images for batches (a) T1 (b) M2_4.9 (c) M3_0.7 show dominantly sheet like structures.

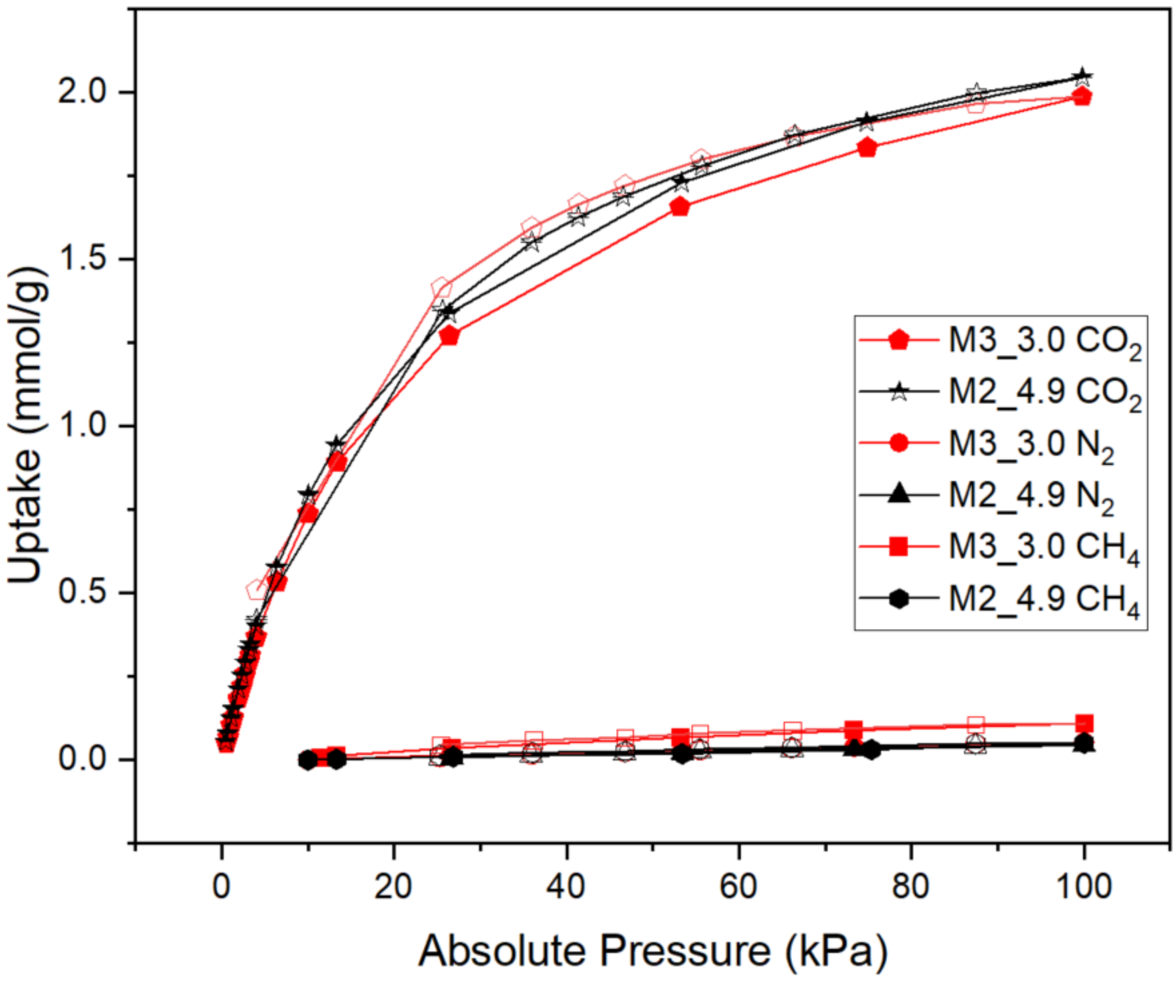

Figure S3: Single component CO_2, N_2 and CH_4 isotherms at 30 °C for batches M2_4.9 (black) and M3_3.0 (red).

Table S1: CO_2 uptakes at 30 °C and 100 kPa

**Batch**	**Uptake (mmol/g)**
R1	0.99
R2	1.30
R3	1.03
R4	0.51
T1	1.65
T2	0.60
S1	1.97
S2(I)	0.66
S2(II)	2.09
S3	1.83
M3_3.0	1.99
M3_0.7	1.92
M3_0.4	1.95
M3_0.2	2.04
M2_4.9	2.05
M2_2.4	2.12
M2_1.6	1.89
M2_0.9	1.99
M2_0.5	0.73
MUF-16(Mn)	2.05
MUF-16(Co/Mn)	1.99

Section 2. SCXRD

Single crystal XRD analysis was performed for one high performing batch (S2(II), CO_2 uptake of 2.09 mmol/g at 100 kPa @30º C) and one low performing batch (R3, CO_2 uptake of 1.03 mmol/g at 100 kPa @30º C). In both cases, the resolved structures have similar unit cell parameters.

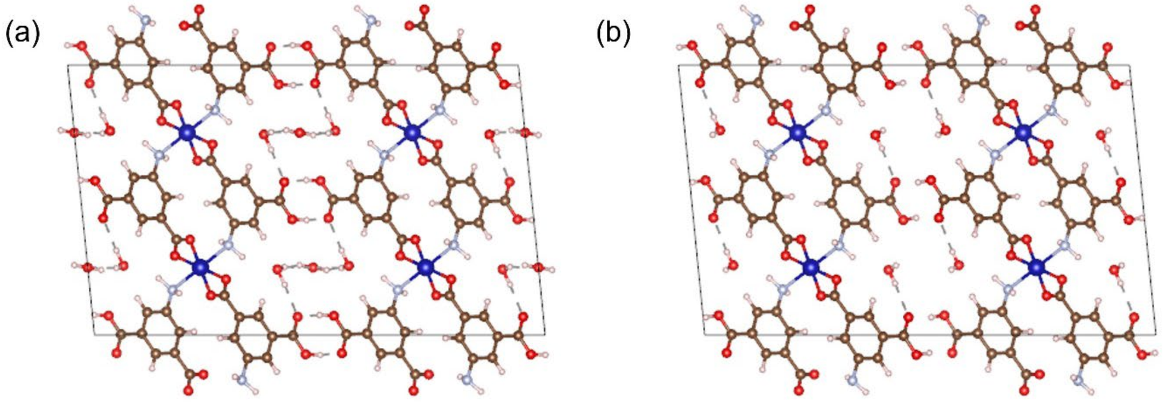

Figure S4: Structures resolved from SCXRD analysis for batches (a) R3 (b) S2(II).

Table S1 shows SCXRD structure parameters for batches R3 and S2(II) and the structure reported by Qazvini et al. [1] (CCDC 1948901). Both the batches synthesized in this work show agreement with each other and with the structure reported in literature.

These structures are reported as CIF files in the “Structure Files” folder in the supplementary information.

Table S2: Space group and unit cell parameters obtained from SCXRD analysis for batches R3, S2(II) and as reported by Qazvini et al.

	**Batch R3**	**Batch S2(II)**	**Qazvini et al. [1]**
Space group	I 2/a	I 2/a	I 2/a
Volume (Å^3)	1731.15	1719.75	1734.37
a (Å)	15.30	15.27	15.35
b (Å)	4.47	4.46	4.42
c (Å)	25.46	25.40	25.61
α (º)	90	90	90
β (º)	95.68	95.61	94.29
γ (º)	90	90	90

Section 3. Thermogravimetric Analysis

TGA curves for multiple batches are given in Figure S3. High performing batches (S1, S2(II), S3 and Mn containing batches) seem to have a relatively higher decomposition temperature (≥350 ºC) while T2, R2 and R3 exhibit a significant loss in weight below 350 ºC (R1 shows the decomposition step at a higher temperature). This indicates that high performing batches in general have slightly better thermal stability. At 350 ºC, about 80 weight percent of the material leaves as gaseous by-products of combustion, leaving metal oxide as residue. The ~80% weight loss is in line with our expectations since stoichiometrically the MOF contains about 14 wt% Cobalt.

However, a clearer indication of the possible presence of loosely bound species in the low performing batches comes from looking at the weight profile between 200 – 350 ºC. Batches S1, S2(II), S3, Mx_y (except M2_0.5) show high CO_2 uptakes and also show a sharp decline in weight at the decomposition temperature. However, the low performance batches show a more gradual mass decline starting at about 200 ºC.

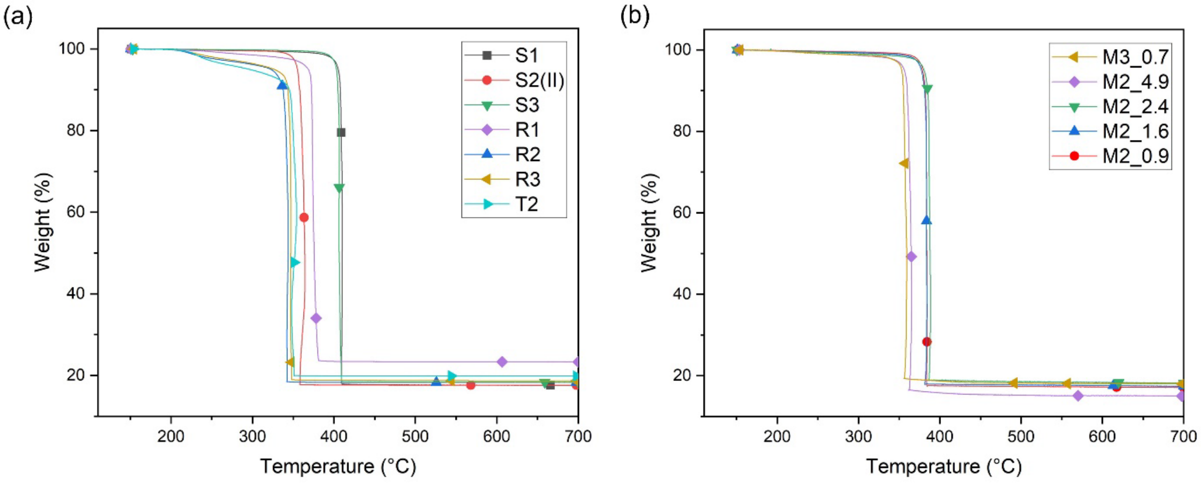

Figure S5: TGA analysis for (a) batches from R, S and T series (b) batches from Mx_y series. The weight percentage is calculated based on dry weight after activating in-situ at 150 ˚C for 2 hours.

Section 4. X-ray Photoelectron Spectroscopy

Cobalt XPS spectra for some additional batches are given in Figure S5. Batches S2(II). T1 and R2 do not show a prominent Co 2p3/2 satellite. M2_0.5, the only low performing batch with Mn, also shows the feature.

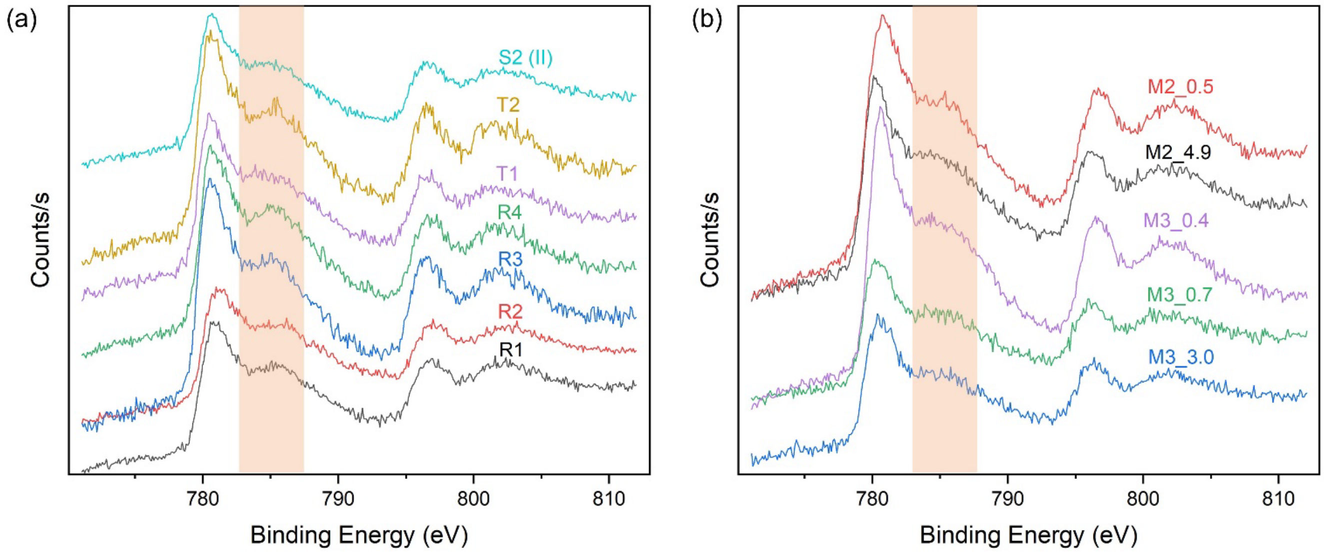

Figure S6: X-ray photoelectron spectroscopy highlighting (orange band) the Co 2p3/2 satellite for (a) Series R, S and T. Batches S2(II) and T1 show CO_2 uptakes > 1.5 mmol/g at 100 kPa at 30 ºC. Batch R2 shows an uptake of 1.3 mmol/g at those conditions. (b) MOF batches with addition of Mn ions where Mx_y indicates addition of Mn^{x+} ions at a concentration of y mmol Mn^{x+}/mol Co^{2+}. All batches except M2_0.5 show high uptake performance.

XPS does not detect the presence of Manganese in the Mx_y series. However, MUF-16(Co/Mn) shows the Mn can be inserted into the MOF backbone (Figure S6). The atomic percentage for MUF-16(Co/Mn) is given in Table S2. Co:Mn ratio is 1.3.

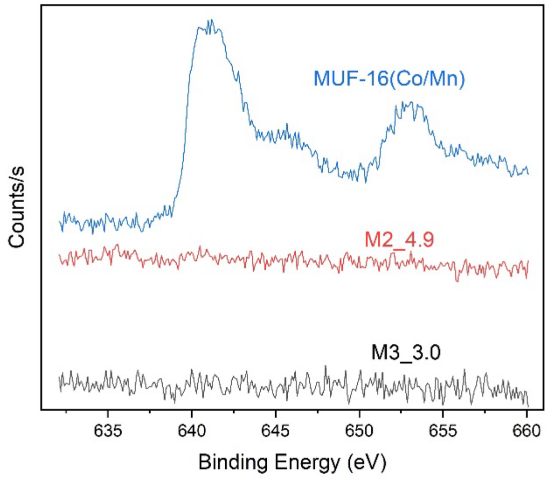

Figure S7: X-ray photoelectron spectroscopy for Mn 2p for batches M2_4.9, M3_3.0 and MUF-16 (Co/Mn). Manganese is not detected in M3_3.0 and M2_4.9. MUF-16(Co/Mn) was synthesized with 1:1 molar ratio of Co and Mn in the reaction mixture.

Table S3: Compositions from XPS of batch MUF-16 (Co/Mn)

	**Atomic %**
O1s	27.05
C1s	60.69
N1s	8.42
Mn2p	1.54
Co2p	2.02

Section 5. FTIR-ATR

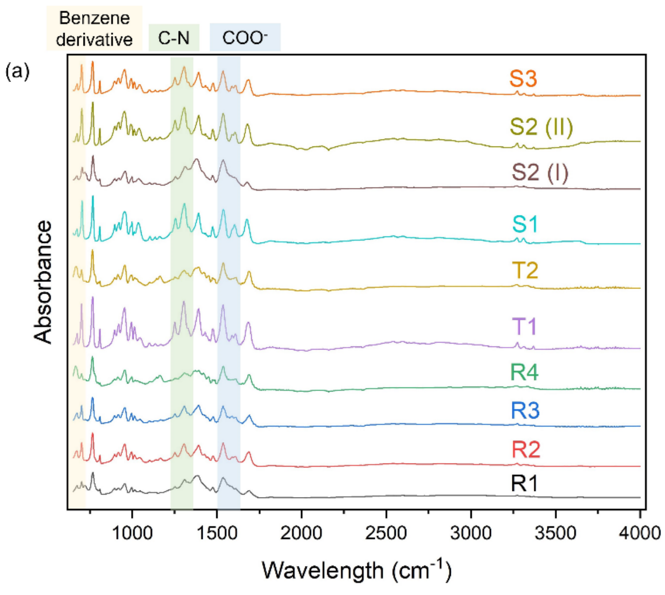

Figure S8: FTIR-ATR spectra for (a) Series R, S and T. Batches S1, S2(II), S3 and T1 show CO_2 uptakes > 1.5 mmol/g at 100 kPa at 30 ºC. (b) MOF batches with with addition of Mn ions where Mx_y indicates addition of Mn^{x+} ions at a concentration of y mmol Mn^{x+}/mol Co^{2+}. All batches except M2_0.5 show high uptake performance.

Section 6. UV Vis

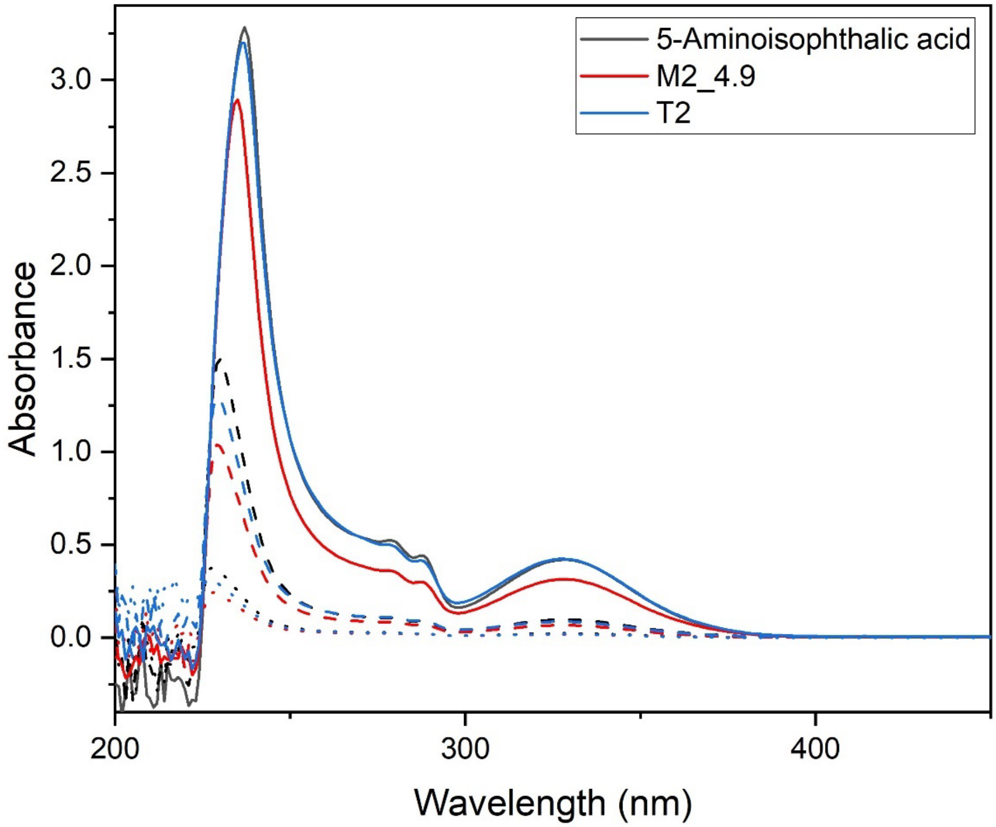

Figure S9: UV Visible absorbance spectra for linker 5-aminoisophthalic acid, batches M2_4.9 and T2. They do not show any difference in solution state. CO_2 uptakes (in mmol/g) of these batches at 100 kPa @ 30 ºC - M2_4.9: 2.04; T2: 0.61. Dashed and dotted lines show successive fivefold dilutions.

Section 7. Energy of mixing calculations

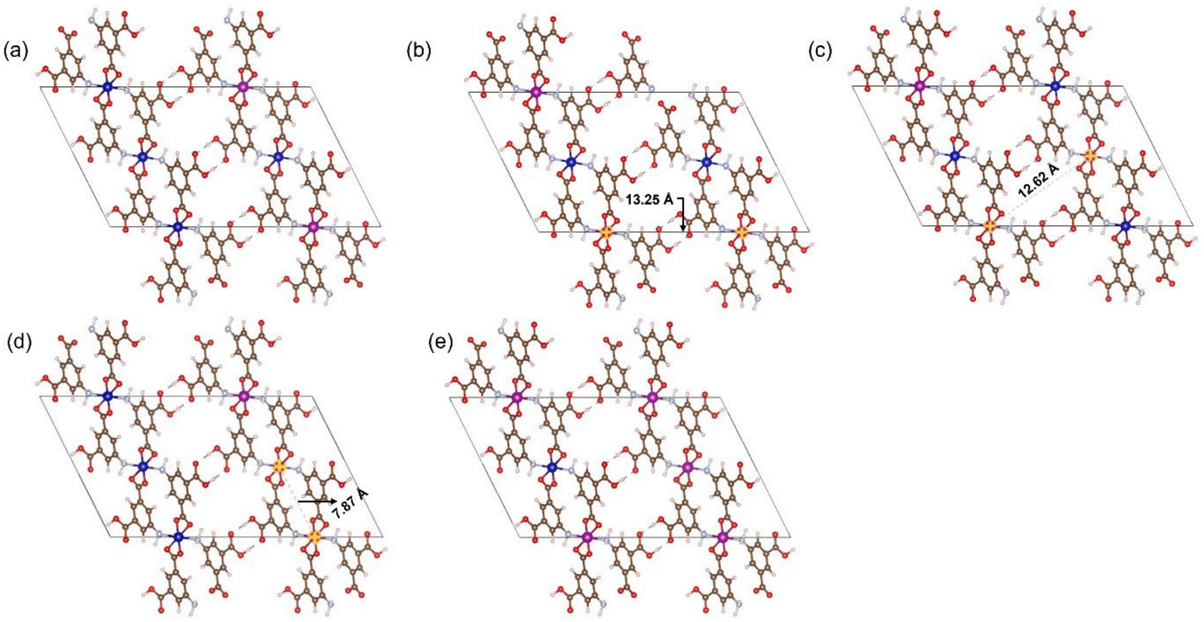

Figure S10: DFT optimized structures containing (a) Co_3Mn_1, (b, c, d) Co_2Mn_2 .The Mn-Mn distances in the structures (b) (c) and (d) are 13.25 Å, 12.62 Å, and 7.87 Å respectively. (e) Co_1Mn_3. Blue = Co, Pink = Mn, Brown = Carbon, Red = Oxygen, Faint Blue = Nitrogen, White = Hydrogen.

These structures are reported as CIF files in the "Structure Files" folder in the supplementary information.

Section 8. Proposed mechanism for impact on Mn on MOF crystallization

A potential mechanism for the apparent catalysis by Mn^{2+} ions of Co-MOF formation is shown below. The MUF-16(Co) structure is represented here by a two-dimensional trimeric fragment. Mn^{2+} undergoes ligand substitution significantly faster than Co^{2+}, although both metal ions are classified as substitution-labile. [2] On this basis we suggest that a binuclear intermediate (structure **1**) can be formed rapidly by two successive steps at the Mn^{2+} center. The last step to form the "complete" MOF, is essentially a carboxylate ligand exchange reaction, assisted for Co^{2+} (which normally undergoes dissociative ligand substitution) by the compensatory delivery of the MOF-associated ligand by Mn^{2+} (which favors associative ligand substitution). [2] We further suggest that Mn^{3+}, which also serves as a catalyst for formation of the complete MUF-16(Co) structure, is reduced to the active Mn^{2+} by Co^{2+} ions, as that process is very strongly driven (by more than 3.0 V).

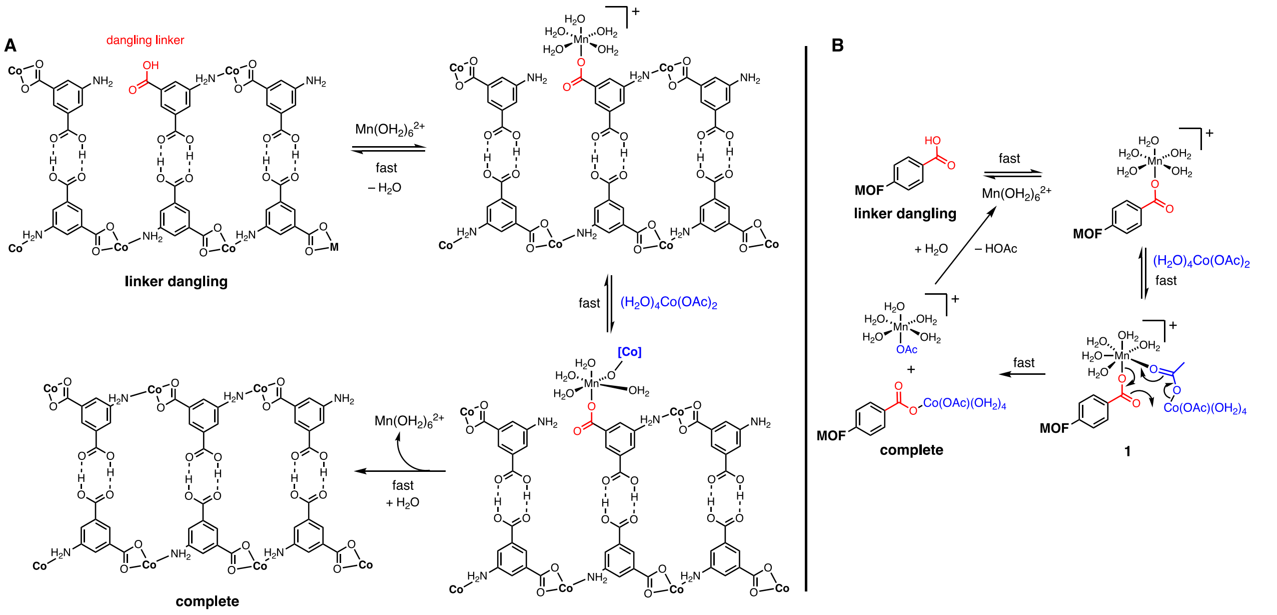

Figure S11: (A) Overall scheme proposed for Mn^{2+}-accelerated elimination of defects in the MUF-16(Co) structure. (B) An example of binuclear Mn-mediated exchange of carboxylate ligands required for this operation.

References:

1. Qazvini, O.T., R. Babarao, and S.G. Telfer, *Selective capture of carbon dioxide from hydrocarbons using a metal-organic framework.* Nature Communications, 2021. **12**(1): p. 197.
2. Richens, D.T., *Ligand substitution reactions at inorganic centers.* Chem Rev, 2005. **105**(6): p. 1961-2002.
